# Generalized Serpiginous Eruption during Immunosuppressive Treatment for Leprosy Reactive Neuritis

**DOI:** 10.1371/journal.pntd.0001357

**Published:** 2011-12-27

**Authors:** Carlos Gustavo Wambier, Fernanda Britta Maitto Lemos, Mark Aaron Cappel, Fernando Bellissimo-Rodrigues, Norma Tiraboschi Foss

**Affiliations:** 1 Division of Dermatology, Department of Internal Medicine, Faculty of Medicine of Ribeirao Preto, University of Sao Paulo, Ribeirao Preto, Brazil; 2 Faculty of Medicine, University of Ribeirao Preto, Ribeirao Preto, Brazil; 3 Department of Dermatology, Mayo Clinic Florida, Jacksonville, Florida, United States of America; 4 Department of Social Medicine, Faculty of Medicine of Ribeirao Preto, University of Sao Paulo, Ribeirao Preto, Brazil; Emory University, United States of America

## Case Presentation

**Figure 1:**
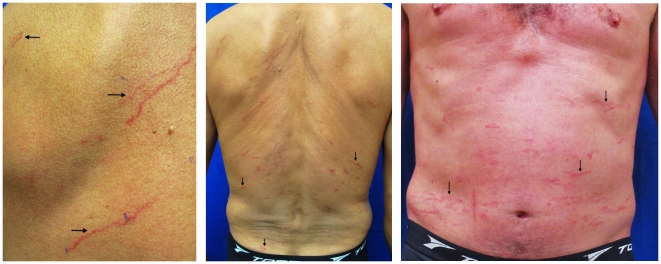
Multiple linear urticarial wheals that expanded serpiginously at approximately 1 cm/15 min, resulting in various tracks in the back and abdomen.

A 49-year-old male farmer with previous diagnosis and treatment of borderline lepromatous leprosy presented with a pruritic cutaneous eruption, demonstrated in Figure 1. This occurred while being treated with prednisone 60 mg (.8 mg/kg) and azathioprine 50 mg per day for leprosy reactive ulnar neuritis. He had noted worsening of the pruritus over the preceding month. He did not have any symptoms of cough, dyspnea, fever, or diarrhea.

Over the past 6 months he had been prescribed various dosages of azathioprine 50–100 mg and prednisone 10–60 mg per day to control relapsing reactive neuritis. His complete blood count revealed frequent eosinophilia and he had negative tests for HIV, hepatitis B and C, and syphilis. He did not have diabetes mellitus.

## Diagnosis

**Figure 2:**
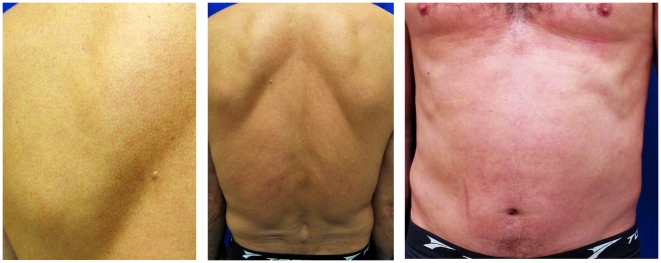
Complete remission after 1 week of treatment. No lesions on the back and abdomen.

**Disseminated larva currens.** *Follow-up and treatment:* Stool parasitology examination revealed *Strongyloides stercoralis* larvae on all three samples. Due to the clinical diagnosis of disseminated larva currens, he was prescribed ivermectin 15 mg for 2 consecutive days (200 µg/kg/day). The prednisone dose was tapered to 20 mg per day. The pruritus resolved and the creeping eruption disappeared in few days after treatment (Figure 2), with no clinical or parasitological recurrence at a 12-month follow-up.

Histopathology of a 4-mm punch biopsy from a lesion on the patient's left shoulder demonstrated a mild mid-dermal perivascular lymphocytic inflammatory infiltrate with rare eosinophils, but no larvae were identified in the sections examined.

## Discussion

This case illustrates the exuberant cutaneous manifestations of larva currens and highlights the importance of primary and secondary prophylaxis of disseminated strongyloidiasis in endemic areas during immunosuppressive treatment such as that used for organ transplantation, oncologic chemotherapy, immunologic diseases, and leprosy reactions. Recently, the initiation of anti-TNF therapy was associated with the exacerbation of the *S. stercoralis* infection in one rheumatologic patient [1]. Although *S. stercoralis* generally causes asymptomatic infection, in the immunocompromised host the number of parasites can increase, leading to autoinfection [2], dissemination, hyperinfection, and death if unrecognized [3].

These chronic recurrent serpiginous eruptions are manifestations of autoinfection by filariform larvae, which are capable of reinfecting the host by penetrating the intestinal wall or by transcutaneous entry points [2], such as the perianal and gluteal area. After reinfection, they disseminate to other organs, including the skin. The autoinfective cycle occurs at a low level throughout infection [2], making larva currens a common, but occasional, phenomenon of few or solitary tracks. However, in an immunocompromised host an accelerated autoinfective cycle may ensue, resulting in generalized pruritic eruption (disseminated larva currens), with multiple and frequent serpiginous tracks [4]. The distinction between autoinfection and hyperinfection is quantitative (parasitological load) and is not strictly defined [2]. Hyperinfection triggers a severe, life-threatening syndrome known as hyperinfection syndrome or “disseminated strongyloidiasis”, which usually presents cutaneous manifestations of vascular injury, such as petechial or purpuric macules [2]. A distinctive sign in hyperinfection syndrome is a periumbilical purpuric macule, known as “the thumbprint sign” [5], [6]. Initial transcutaneous *S. stercoralis* infection may also present acute cutaneous reactions at the site of larval entry, such as lower and upper extremities.

Physicians should be able to make a presumptive clinical diagnosis of larva currens based on the observation of rapidly moving linear or serpiginous tracks. Differential diagnoses include dermographism and cutaneous larva migrans. The authors use pen markings on the extremities of these tracks to easily detect movement, as illustrated in Figure 1. Skin biopsies frequently fail to reveal the rapidly moving *S. stercoralis* [7].

**Key Learning Points**

- Presentation of disseminated larva currens as multiple pruriginous erythematous serpiginous wheals
- Therapeutic immunosuppression is the trigger factor for dissemination in a patient with *S. stercoralis* infestation
- Hyperinfection syndrome and death are possible complications of untreated cases;
- Effective treatment is possible with ivermectin 200 µg/kg/day for 2 days
- Differential diagnosis: cutaneous larva migrans and dermographism—both can be ruled out by detection of movement within minutes by pen markings on the extremities of the tracks on physical examination
